# Presence and activities of clinical research coordinators at Italian Health Care Institutions: A national cross-sectional survey

**DOI:** 10.1017/cts.2021.872

**Published:** 2021-10-28

**Authors:** Caterina Caminiti, Giuseppe Maglietta, Ileana Frau, Giulia Peruzzotti, Mariagrazia Felisi, Antoinette van Dijk

**Affiliations:** 1 Clinical and Epidemiology Research Unit, University Hospital of Parma, Parma, Italy; 2 Site Relationship Manager (SRM) Site Management, IQVIA, Milan, Italy; 3 Clinical Trial Office IEO, European Institute of Oncology IRCCS, Milan, Italy; 4 Quality Assurance Unit, Consorzio per Valutazioni Biologiche e Farmacologiche, Pavia, Italy; 5 Clinical & Medical Affairs Unit, D.O. Research Sagl, Lugano, Switzerland

**Keywords:** Clinical research coordinator, data manager, nonclinical research staff, investigator-initiated studies, research infrastructure

## Abstract

**Background:**

Clinical Research Coordinators (CRCs) are key members of research teams who ensure rigorous conduction of clinical studies and quality standard compliance. Yet, their roles and responsibilities are still not well defined, and formal recognition of their professional profile is lacking in Italy. This survey of Italian healthcare institutions collected data on centers’ research activities number of CRCs and tasks they performed and explored factors associated with CRC employment.

**Methods:**

Cross-sectional study using a brief questionnaire. Data were analyzed by means of graphical representations, histogram, scatter, and polar plots. Multivariable linear regression was specified to test the association between the number of CRCs and a subset of factors.

**Results:**

Data collection took place from February to December 2020. 62/143 institutions (43.4%) responded. Median number of ongoing studies reported by centers was 65 (IQR 29–205); of these, median of sponsored and interventional studies was 32 and 35, respectively. Median number of CRCs employed at each center was 6 (IQR 2–9). The frequency with which activities were reported to be performed by CRCs overlapped with those of Data Managers. Linear multivariable regression analysis revealed a statistically significant association between the number of employed CRCs and the number of sponsored studies (*P* = 0.01), but not with the total number of studies, geographical location, or institution type.

**Conclusions:**

The association between industry funding and the number of CRCs observed in this study should be further explored to understand the direction of this relationship and to verify whether this may influence compliance with quality standards.

## Introduction

The clinical research environment is becoming more complex and highly competitive. More and more clinical trials are now multicenter, often multinational, and involve the collection and analysis of large amounts of data. In addition, their conduction is complicated by stringent legal and regulatory requirements, which can imply a heavy administrative burden [1,2]. No single individual could be expected to fulfill all the tasks required to achieve high-quality research; therefore, the success of a quality clinical research program requires an exemplary research team comprising different competencies [3]. Clinical Research Coordinators (CRCs) are key members of a research team. They closely interact with the Principal Investigators and research staff, sponsors and/or contract research organizations, patient advocacy groups, ethics committees, and national agencies regulating drug development, to ensure that studies are carried out rigorously and in compliance with Good Clinical Practice (GCP) requirements [4-6]. Their contribution is also essential to the timely and successful translation of pharmaceuticals and medical devices into clinical applications to improve human health [7]. The need for CRCs in clinical research teams has recently been emphasized in the literature. For example, in a 2017 web survey conducted on 319 Italian cancer centers (response rate 115/319 = 36%) aimed at mapping the composition and the organization of research teams [8], contribution of CRCs was judged essential for trial conduct in 82.4% of responders, and 83.3% responded that the quality of clinical research had absolutely improved after a CRC became a member of the clinical research team. Also, activities generally performed by CRCs are considered essential by decision-makers for trial site selection, as suggested by a survey involving 20 biopharmaceutical companies and 23 Clinical Research Organizations [9]. When asked what they would prefer that trial sites be best at, respondents attached priority to having the first patients ready for inclusion (42%) and to having good data entry, documentation, and reporting practice (25%), followed by easily reachable site personnel and backup (23%). Despite the crucial importance of CRCs in the clinical research community, the roles and responsibilities of these professionals remain unclear, which is also reflected in the many different job titles used for them (data manager, study coordinator, project manager, and research assistant). In Italy in particular, the English terms “study coordinator” and “data manager” are often used interchangeably to indicate a nonclinical professional dedicated to supporting the Principal Investigator, ensuring data accuracy and GCP compliance [6]. This lack of clarity presents challenges to those functioning in a CRC role. CRCs responding to surveys carried out in different countries, including Europe, North America, and Latin America, report lack of professional identity, inadequate remuneration, and lack of peer support and recognition [10,11]. In Italy in particular, the CRC professional profile is not formally recognized by specific certifications, and no formal CRC position exists in institutions’ staff, as required by national health care workforce regulations. This makes it difficult for institutions and policymakers to appraise the demand for CRCs and to respond by allocating the necessary resources.

This study addresses this gap by collecting and analyzing data from Italian health care institutions, with the aim to describe their research activities in 2019 in terms of the number of ongoing studies and the presence of CRCs. It also aimed to investigate which of the explored factors were associated with the presence of CRCs in clinical centers.

This survey was conceived by the Italian Association of Contract Research Organizations (AICRO), in collaboration with the Federation of Associations of hospital Internists, the Data Manager Italian Group, and the Federation of Italian Cooperative Oncology Groups. These entities share a long-standing commitment to improving the recognition of the professional role of CRCs and their contribution as an essential component of institutions’ research teams. The initiative was coordinated by AICRO’s Clinical Trial Center Working Group (WG-CTC).

The study is reported following the Strengthening the Reporting of Observational Studies in Epidemiology guidelines and its extension for cross-sectional studies [12].

## Materials and Methods

### Study Design, Setting, and Participants

This was a cross-sectional study conducted through a paper questionnaire that was addressed to Italian health care institutions (University Hospitals, Community Hospitals, and Scientific Research Institutes) selected from the Ministry of Health’s Database, which contained 550 facilities. To build a representative and exhaustive sample of research contexts nationwide, the following criteria were applied in the selection of the institutions:

- all Scientific Research Institutes (Istituti di Ricovero e Cura a Carattere Scientifico − IRCCS)
- all centers certified to conduct a Phase I studies according to Italian regulations
- at least one cancer institution per Region, where available
- at least one pediatric institution per Region, where available
- facilities had to have hosted at least one clinical trials in the past year.

Overall, at least 10% of centers of each region had to be included; this proportion was considered sufficient to ensure geographical representativeness without undermining feasibility. Sample size was not based on a priori statistical considerations.

### Study Procedures

To enhance adherence an invitation letter was sent by AICRO to the General Managers of selected Institutions, presenting the initiative and asking for support to its conduction. Centers were divided into groups and each group assigned to a member of AICRO’s WG-CTC, who became its reference person, in charge of coordinating data collection and ensuring communication with the centers. The referring persons identified research staff working in the institutions’ research Offices/Departments, to whom they explained the study in detail and, if necessary, provided indications on how to organize data collection within their center. Time allowed for data gathering was max 6 months, to provide data reflecting the current context. Responses were collected from centers on paper forms and then entered into a database by AICRO staff.

The questionnaire investigated the number of Data Managers (DMs)/CRCs employed at the center, and the number of ongoing studies (any research approved by the Ethics Committee) in 2019, requiring to specify how many of these studies were investigator initiated, sponsored by the pharmaceutical industry, interventional, and coordinated by the center. The questionnaire also listed 13 research activities, asking centers to indicate whether each activity was performed by a DM or CRC. Information on other staff members working at the center and related activities was also collected, but it is not reported here because either not pertinent to the aims of this paper or with a significant amount of inconsistencies/missing data. As no similar surveys were available, the set of information to be collected was determined empirically prioritizing ease of questionnaire completion.

This survey only collected data in aggregate form without any reference to patients or individual staff members; therefore, Ethics Committee approval was not sought and informed consent was not necessary.

### Statistical Methods

Descriptive statistics, as absolute and relative frequencies, median, and Interquartile Range, were used to summarize data. Graphical representations, box, and polar plots were constructed to illustrate the distribution of specific variables and to detect potential outlier values. Correlation matrix was generated to explore potential links between study variables. Multivariable linear regression was specified to test the association between the number of CRCs (dependent variable) and a subset of factors collected by the questionnaire (independent variables): center location, institution type, number, and type of ongoing studies. Data were analyzed using R software v. 4.0.1.

## Results

Data collection took place from February 2020 to December 2020. One hundred and forty-three institutions were contacted, of which 62 responded (Response rate 43.4%). The characteristics of centers which did and did not respond are shown in Table 1. A statistically significant difference was observed in terms of geographical distribution of respondents, with a lower participation of institutions located in Central and Southern Italy (*χ* ^2^ = 9.3, *P* = 0.002). No statistically significant difference in terms of institution type was detected, although Community Hospitals exhibited lower response rates as compared to University Hospitals and Research Dedicated Centers.

**Table 1.** Center characteristics, responders vs nonresponders

	Respondents	Nonrespondents	Total	*P*-value
Location				
Northern Italy	39 (27.3%)	29 (20.3%)	68 (47.6%)	0.002
Central-Southern Italy	23 (16.1%)	52 (36.3%)	75 (52.4%)	
Total	62 (43.4%)	81 (56.6%)	143 (100%)	
Institution type				
IRCCS	30 (21%)	34 (23.7%)	64 (44.8%)	n.s.
University Hospital	13 (9.1%)	13 (9.1%)	26 (18.2%)	
Community Hospital	19 (13.3%)	34 (23.7%)	53 (37.1%)	
Total	62	81	143 (100%)	

IRCCS, Istituto di Ricovero e Cura a Carattere Scientifico (Scientific Research Institute); n.s, nonsignificant.

**Fig. 1.**
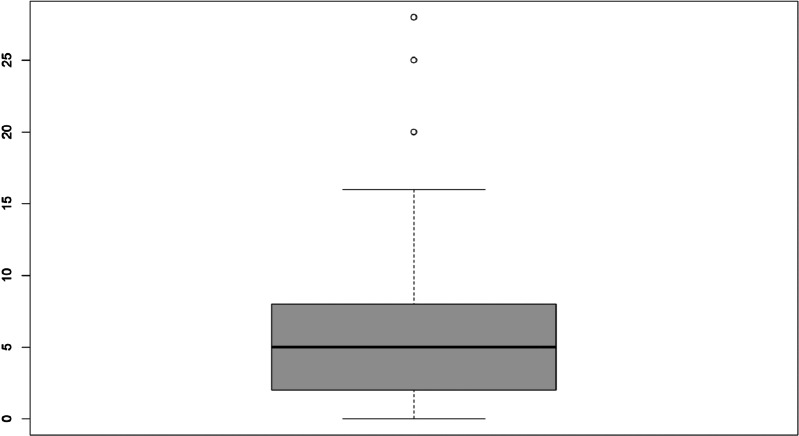
Box plot displaying the distribution of the number of data managers/clinical research coordinators employed in 2019. The box depicts the interquartile range (first to third quartiles) and the median (second quartile, line in bold). Outliers are represented by dots.

Table 2 summarizes questionnaire responses. The median number of ongoing studies reported by centers was 65 (IQR 29–205); of these, the median of sponsored and interventional studies was 32 and 35, respectively. The median number of DMs/CRCs employed in 2019 was 6 (IQR 2–9). As shown in the box plot (Fig. 1), values beyond 9, the third quartile, exhibit wide dispersion indicating that a small number of centers employs a number of DMs/CRCs up to four times the median.

**Table 2.** Summary of responses to survey questions

	Median (Interquartile range, IQR)
Number of ongoing studies per institution	65 (29–205)
of which For Profit	32 (8–79)
of which interventional	35 (10–101)
of which coordinated by the center	0 (0–22)^a^
Number of CRCs/Data managers	6 (2–9)

CRC, clinical research coordinators.

a Available data concern 20 out of 64 responses, of which 10 reported the value 0.

**Fig. 2.**
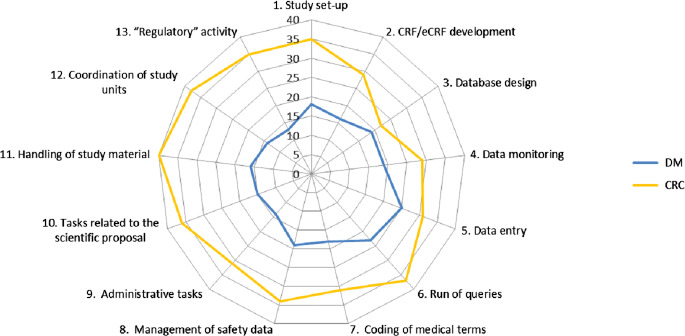
Polar plot representing the frequency with which each of the 13 activities at the centers is performed by the data managers (DM; blue line) or the clinical research coordinators (CRC; yellow line). The furthest lines are from the center, the most frequently activities are performed by the data managers or clinical research coordinator.

Fig. 2 illustrates the frequency with which the 13 activities listed in the questionnaire were reported to be performed by the DMs or CRCs employed at the center. As shown in the polar plot, activities relating to data management (database design, data monitoring, data entry, run of queries) were assigned with similar frequency to DMs and CRCs.

**Fig. 3.**
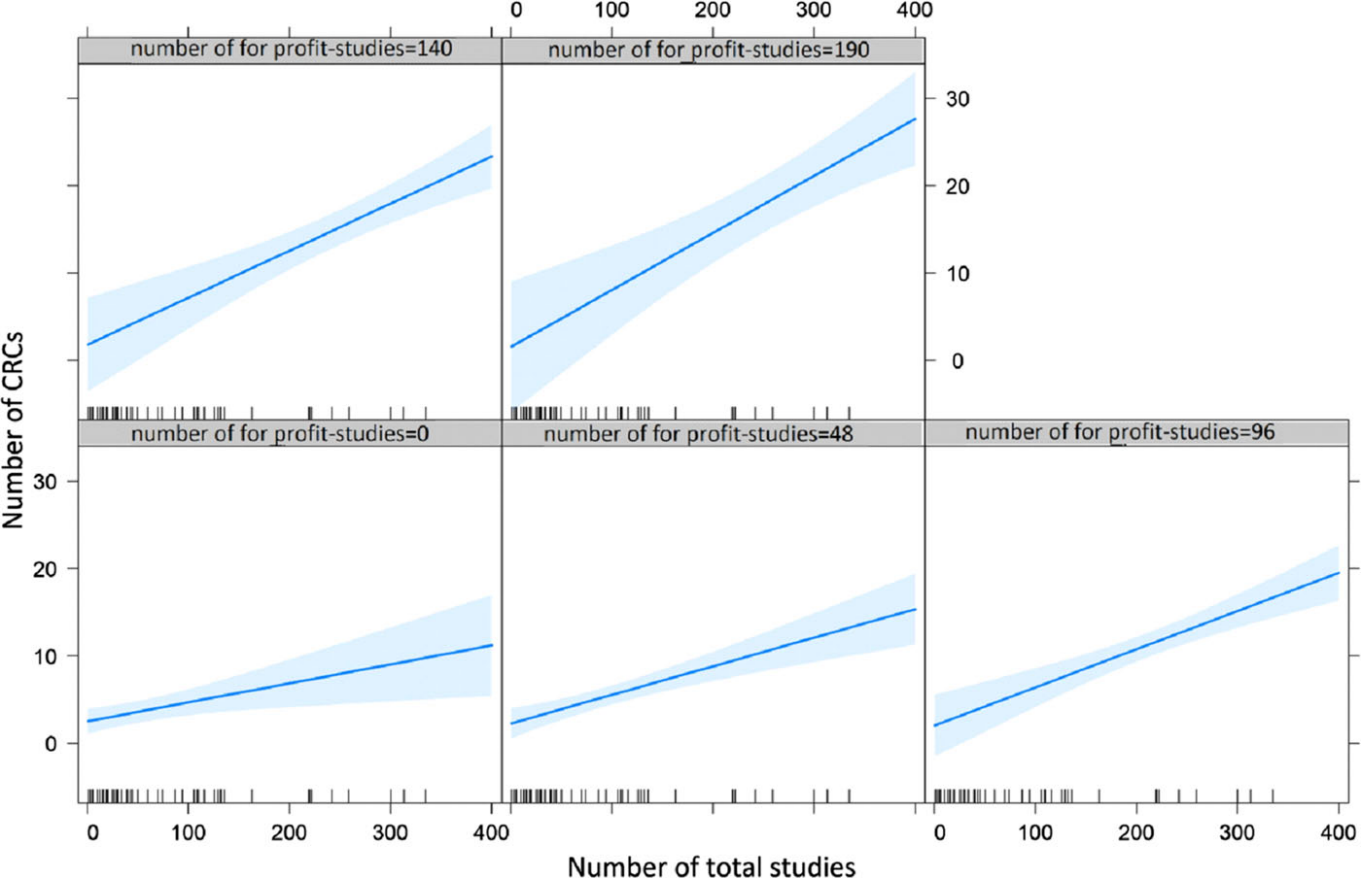
Plots representing the interaction between for-profit and overall studies on the number of clinical research coordinators (CRCs). Each plot depicts the relationship (regression line, and blue area as confidence intervals) between the number of total studies (*X* axis) and the number of CRCs (*Y* axis). The five plots are distinguished by the incremental number of for profit studies.

Multivariable linear regression analysis (Table 3) revealed a statistically significant positive association between the number of CRCs employed at the center and the number of sponsored studies (*P* = 0.01), but no association concerning geographical location and type of institution. Notably, the increase of the overall number of studies does not appear to impact on the number of CRCs, whereas the increase in sponsored studies increases the number of CRCs (Fig. 3).

**Table 3.** Multivariable linear regression analysis for factors associated with clinical research coordinator (CRC) number at clinical centers

	Linear regression coefficient	Standard error	*P*-value
Variables			
Intercept	1.44	0.99	0.16
Total number of studies	0.021	0.008	0.012
For-Profit	0.004	0.02	0.87
IRCCS	0.61	0.81	0.46
North	1.14	0.86	0.19
Total number of studies*For-Profit	0.001	0.0001	0.03

IRCCS, Istituto di Ricovero e Cura a Carattere Scientifico (Scientific Research Institute).

## Discussion

To our knowledge, this is the first survey investigating the presence of CRCs in Italian health care institutions. The main finding of this work is the direct relationship between the number of studies funded by the pharmaceutical industry (for-profit) and the number of CRCs employed at the center, regardless of institution type (University Hospital, IRCCS, Community Hospital) or location. These findings, not previously reported, may also be due to the lack of public funding for clinical research in Italy, which is below the EU average, and further below the USA [13]. However, the cross-sectional nature of this study does not enable to ascertain the direction of this relationship. In fact, it could be hypothesized that sponsors are more likely to select sites with CRCs, but also that CRCs are more likely to be hired once funding is received. Research is also warranted to assess whether the presence, or absence, of CRCs has an impact on compliance with quality standards.

The above-mentioned survey by Cinefra et al. [8], similar to ours but only restricted to cancer centers, also found a direct association between the number of clinical studies (experimental and observational) and the number of CRCs (*P* = 0.0016): in all the sites with >50 active trials, at least three CRCs were present. However, the influence of the nature of sponsorship was not reported.

Notably, in our survey the number of CRCs did not appear to be influenced by the type of institution. This is surprising, as we expected to find more CRCs employed in hospitals with high research volumes, especially in IRCCS, which are highly specialized centers funded by the Italian Ministry of Health specifically for research purposes. These findings are not in line with those of the Cinefra survey, where CRCs were more frequently employed at IRCCS: 72.2% of IRCCS employed three or more CRCs, compared to 21.4% and 17.2% of Community and University Hospitals, respectively (*P* = 0.0023). This may be due to the fact that the Cinefra survey, unlike ours, only included oncology IRCCS, where a substantially higher number of studies is funded by the pharmaceutical industry.

Our survey confirms that activities performed by CRCs are diverse and sometimes overlap with those of other professionals, especially the data manager. This “confusion” is not limited to Italy. The literature emphasizes that capacity development for clinical research is held back by a lack of recognition for the skills acquired through involvement in research and that core roles within clinical research are little understood and still relatively unrecognized as viable career paths [14]. Similarly, a survey on support provided to CRCs at member institutions of the National Institutes of Health’s Clinical and Translational Science Award consortium [15] emphasized a major gap in the ability to identify and communicate with CRCs. Fewer than half of the 78 responding institutions had a complete (or “fairly complete”) database for contacting RCs, and 96% indicated that RCs operated under many different titles. This diversity of roles is an important factor to consider when identifying individuals performing the tasks of a CRC [15]. Attempts have been made toward clarifying the competencies for specific clinical research roles, such as by the Special Programme for Research and Training in Tropical Diseases (TDR) of the World Health Organization [14] and by the Multi Regional Clinical Trials Center at Harvard University [16]. A competence assessment tool specific for the CRC role, called CICRP-II, has also been validated in a work supported by the US National Institutes of Health [7]. However, no standardization still exists; guidance such as the GCPs and the new EU regulation on clinical trials [17] only give general indications on the necessary characteristics of research personnel. Some roles have received more attention than others. In Italy in particular, the CRC appears to be neglected by current regulations, unlike other professional profiles, such as the Clinical Research Associate, Quality Assurance and Auditor, for whom requirements are set forth in national legislation (Ministerial Decree 15 November 2011).

This study has some limitations. First, as data were self-reported, the possibility of response bias cannot be excluded. Respondents may offer biased responses for various reasons. For example, they might want to present reality in a desirable way, to “look good” [18]. In our survey, information was declared and reported by a reference person for research at each institution. We cannot rule out that these individuals may have provided information in such a way to exhault their center’s research performance, or vice-versa to denounce the lack of dedicated personnel despite an intense research activity. Another possible source of bias may be related to mere misunderstanding [18]. Indeed, the high proportion of inconsistencies/missing data for some responses suggests that corresponding questions may have been misinterpreted. For example, when asked about the presence of research nurses, residents and PhD students, sometimes the total number of these professionals employed at the center was indicated, not restricted to those involved in research. These issues, however, did not concern data on CRCs, which are the subject of this paper. Finally, asking participants to state information in retrospect makes responses susceptible to recall bias [18]. Second, institutions were included applying criteria aimed to identify the centers most likely to conduct clinical research. Thus, this analysis may not be representative of the current Italian situation. A third limitation is linked to generalizability, as the survey was restricted to Italian centers and did not use validated tools to describe activities or investigate the actual level of competence of CRCs working at healthcare institutions. This makes it difficult to carry out comparisons with other countries .The data collected by this survey, though purely descriptive, may be valuable in future analyses, also to measure the impact of interventions aimed at the recognition of the CRC role in healthcare institutions [7]. The new EU Regulation on Clinical Trials [17] calls for equal quality standards for research, which should be ensured regardless of the nature of sponsorship. To comply with these indications, it is essential that clinical research teams comprise highly competent figures such as the CRC, also involved in studies conducted by noncommercial sponsors. It is thus hoped that clear, standardized indications are provided at a European level on how institutions can determine the need for CRCs, on the competencies and expertise required to fulfill their roles, and on career opportunities that should be offered to these professionals. This would both grant CRCs well-deserved recognition and greatly benefit the quality of research, ultimately to the advantage of patient health.
